# Individualized perfusion targets in hypoxic ischemic brain injury after cardiac arrest

**DOI:** 10.1186/s13054-017-1832-9

**Published:** 2017-10-24

**Authors:** Mypinder S. Sekhon, Donald E. Griesdale

**Affiliations:** 1 Department of Medicine, Division of Critical Care Medicine, Vancouver General Hospital, West 12th Avenue, University of British Columbia, Vancouver, BC V5Z 1M9 Canada; 2 Department of Anesthesiology, Pharmacology and Therapeutics, Vancouver General Hospital, West 12th Avenue, University of British Columbia, Vancouver, BC V5Z 1M9 Canada; 3 Centre for Clinical Epidemiology and Evaluation, Vancouver Coastal Health Research Institute, 899 West 12th Avenue, University of British Columbia, Vancouver, BC V5Z 1M9 Canada; 4 Critical Care Medicine, Vancouver General Hospital, Room 2438, Jim Pattison Pavilion, 2nd Floor, 855 West 12th Avenue, Vancouver, BC V5Z 1M9 Canada

**Keywords:** Hypoxemic ischemic brain injury, Cardiac arrest, Cerebral autoregulation, Mean arterial pressure, Cerebral oxygen delivery, Secondary injury

## Abstract

Secondary injury is a major determinant of outcome in hypoxic ischemic brain injury (HIBI) after cardiac arrest and may be mitigated by optimizing cerebral oxygen delivery (CDO_2_). CDO_2_ is determined by cerebral blood flow (CBF), which is dependent upon mean arterial pressure (MAP). In health, CBF remains constant over the MAP range through cerebral autoregulation. In HIBI, the zone of intact cerebral autoregulation is narrowed and varies for each patient. Maintaining MAP within the intact autoregulation zone may mitigate ischemia, hyperemia and secondary injury. The optimal MAP in individual patients can be determined using real time autoregulation monitoring techniques.

## Introduction

Cardiac arrest leads to immediate cessation of cerebral blood flow (CBF) and secondary injury after return of spontaneous circulation (ROSC) [1]. This cerebral insult, termed hypoxic ischemic brain injury (HIBI), is the primary determinant of outcome [2]. Despite timely ROSC, ongoing cerebrovascular dysfunction can lead to secondary injury and worse neurologic outcome. HIBI management focuses on mitigating this secondary injury by optimizing blood glucose, temperature and, importantly, the balance between cerebral oxygen delivery (CDO_2_) and utilization [3].

CDO_2_ is dependent upon CBF, which in turn is principally determined by mean arterial pressure (MAP). Recognizing the lack of high quality data, the American Heart Association recommends maintaining a MAP ≥ 65 mmHg and systolic blood pressure ≥ 90 mmHg after ROSC [4]. These uniform hemodynamic thresholds fail to account for individual variability within patient and disease pathophysiology. Ultimately, this “one-size fits all” approach may subject HIBI patients to secondary ischemic injury and worse outcome [5].

Cerebral autoregulation, through compensatory vasodilation and vasoconstriction, is the innate ability of the cerebrovasculature to maintain stable CBF over a wide range of MAP [6]. In health, autoregulation ensures the cerebral parenchyma is protected from both ischemia and hyperemia. At the cellular level, endothelial release of vasodilators and vasoconstrictors as well as activation of L-type calcium channels with signaling from adjoining astrocytes are responsible for the observed CBF homeostasis in response to fluctuations in arteriole blood pressure [7]. Historically, complete loss of autoregulation was thought to occur after brain injury, resulting in a linear relationship between CBF and MAP [8]. In fact, autoregulation may remain intact, albeit with a narrowed and right-shifted intact zone after HIBI [9]. Importantly, the specific zone of intact autoregulation appears to have significant heterogeneity between individual patients after HIBI [9, 10]. Our ability to detect the precise MAP threshold at which autoregulation occurs may eventually lead to personalized perfusion targets after cardiac arrest. With this in mind, the purpose of this viewpoint is to discuss the advances in autoregulation monitoring in patients with HIBI. Furthermore, we discuss how this technology may dramatically shift our thinking from the traditional fixed MAP thresholds to personalized perfusion targets.

## Methods

In this viewpoint, we conducted a non-systematic literature search on MEDLINE and EMBASE to identify pertinent citations which were referenced in our study. We did not examine conference abstract proceedings. Subsequently, study abstracts were reviewed and manuscripts describing autoregulation monitoring techniques after cardiac arrest were used as references.

## Discussion

The acute post resuscitative phase of HIBI is characterized by a “no reflow” phenomenon resulting in persistent cerebral oligemia despite restoration of cardiac output. No reflow, in part, stems from diffuse cerebrovascular inflammation, intravascular microthrombi [11], dysfunctional nitric oxide signaling and perivascular cerebral edema culminating in increased cerebrovascular resistance and decreased CBF [12]. Hypotension, particularly below the lower limit of autoregulation, during this critical period may further exacerbate ongoing cerebral ischemia and secondary injury [13]. Trzeciak and colleagues retrospectively demonstrated an association between hypotension (systolic blood pressure < 90 mmHg) and increased in-hospital death (OR 2.7 [2.5–3.0]) in 8736 cardiac arrest patients [14]. Furthermore, Laurikkala and colleagues prospectively found an association between unfavorable neurological outcome and time spent *below* a MAP of 70 mmHg in the first 48 h after ROSC [15]. Multiple additional observational studies demonstrate associations between hypotension after ROSC and poor neurologic outcome and death [14, 16–21]. Our research group performed a systematic review and concluded that improved neurologic outcomes after cardiac arrest are associated with increased blood pressure, albeit with significant between-study heterogeneity [5].

Although uniformly increased MAP targets seem intuitive to mitigate secondary injury in HIBI and are supported by emerging observational literature, guideline statements recommend a MAP threshold of ≥ 65 mmHg [4]. The persistence of this universal MAP threshold in guidelines is in part driven by the lack of large randomized control trial data. Recently authors have argued to move beyond the question of uniformly increased MAP versus current guideline-based MAP targets and instead focus on individualized hemodynamic thresholds in the setting of post-cardiac arrest patient physiology and pre-existing comorbidities [22, 23].

Cerebral autoregulation monitoring allows us to determine individualized MAP thresholds, thereby moving towards a personalized resuscitative strategy. Sundgreen and colleagues conducted a sentinel prospective physiologic study to assess autoregulation in patients after cardiac arrest using transcranial Doppler to assess middle cerebral artery blood flow velocity while incrementally augmenting MAP with intravenous norepinephrine [9]. There was considerable between-patient variability in the zone of intact autoregulation. In a third of patients, the zone of autoregulation was markedly narrowed and right shifted above a MAP of 100 mmHg [9]. In the remaining patients, nearly half demonstrated a complete loss of autoregulation, highlighting the marked heterogeneity in cerebrovascular hemodynamics between HIBI patients [9]. Although transcranial Doppler-based autoregulation assessment is well established, inter-observer reliability, adequate middle cerebral artery insonation windows and the inability to perform long-term continuous monitoring limit its widespread use. Therefore, alternative methods of autoregulation monitoring are required.

**Fig. 1:**
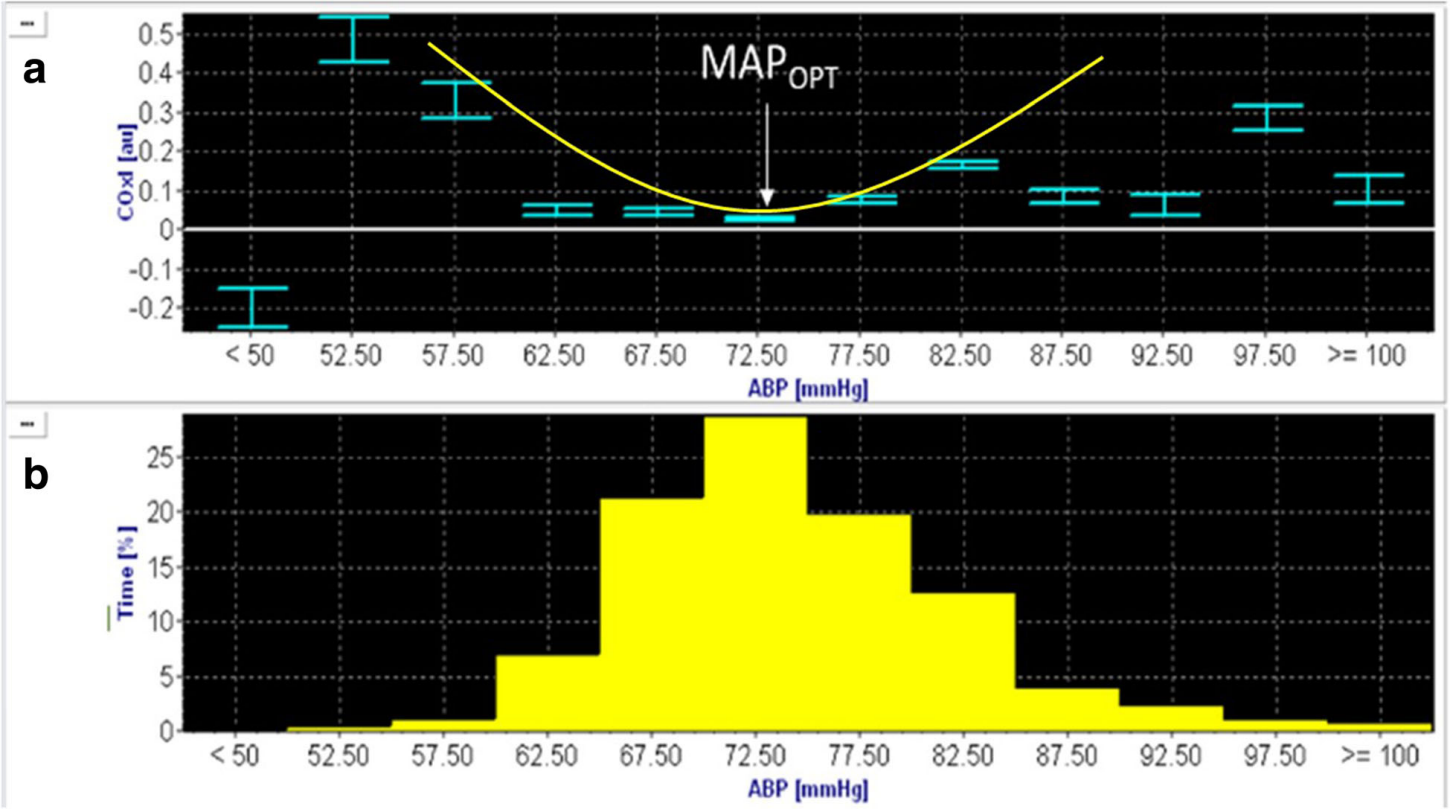
Autoregulation-based identification of optimal mean arterial pressure after hypoxic ischemic brain injury. **a** A best fit U-shaped curve with the nadir of the curve representing the optimal mean arterial pressure (*MAP*_*OPT*_). The COx is plotted along the y-axis and mean arterial pressure in 5-mmHg bins on the x-axis, denoted as *ABP*. **b** The amount of time during the monitoring period spent within each 5-mmHg bin of mean arterial pressure. The total duration of time is denoted as a percentage on the y-axis with each 5-mmHg mean arterial pressure bin on the x-axis. This figure is original for this manuscript

Cerebral autoregulation can also be assessed non-invasively using the relationship between MAP and regional saturation of oxygen (rSO_2_) using near infrared spectroscopy (NIRS) [24]. NIRS emits infrared light at frequencies between 700 and 950 mm where is it subsequently refracted or absorbed in the first 1 to 2 cm of frontal lobe tissue and estimates a percentage of a ratio of oxygenated versus deoxygenated hemoglobin, termed the (rSO_2_) [25]. To assess autoregulation, we can observe how fluctuations in MAP will result in changes to rSO_2_. When autoregulation is compromised, rSO_2_ is positively correlated with MAP (e.g., increasing MAP leads to increasing rSO_2_) [24]. Conversely, when autoregulation is intact, rSO_2_ remains constant despite fluctuations in MAP [24]. Over time, a moving correlation coefficient between MAP and rSO_2_ can be calculated, termed COx. A positive COx indicates dysfunctional autoregulation [26] and a negative or near zero COx indicates intact autoregulation [26]. Generally, autoregulation is considered to be preserved when the COx is less than 0.3 [27]. After plotting COx (y axis) versus the individual patient’s MAP range, a U shaped curve can be produced and the nadir of this curve corresponds to the optimal MAP for each individual patient (Fig. 1) [28]. (Figure 1 is original for this manuscript and was used with approval from the University of British Columbia clinical research ethics board—H15-01606.)

COx-based autoregulation monitoring following cardiac arrest is well studied in pediatric swine models of neonatal HIBI. As part of these animal studies, Brady et al. established that COx was able to accurately detect the lower limit of autoregulation with a sensitivity of 92% and receiver operator characteristics area under the curve of 0.89. Furthermore, these authors also demonstrated that the identification of the lower limit of autoregulation using COx was in agreement compared to invasive monitoring techniques using laser Doppler flow and pressure reactivity index [29].

Clinically, Howlett et al. [30] have shown that neonates who spent a greater proportion of time below the optimal MAP had an increased burden of basal ganglia, white matter and thalamus injuries after HIBI. Similarly, Lee and colleagues have shown that deviation from the identified optimal MAP using COx was associated with magnetic resonance imaging features of HIBI in neonates after perinatal asphyxia [31]. These authors have also shown that deviation from optimal MAP was associated with poor neurologic outcome in 36 pediatric cardiac arrest patients [32]. Burton et al. [33] confirmed these results by studying 28 neonates with HIBI after asphyxia and found worse motor and cognitive function in those patients who had a greater time of deviation below the identified optimal MAP with NIRS-based autoregulation assessment.

In adult cardiac arrest subjects, Ameloot and colleagues retrospectively calculated COx and demonstrated that cerebral autoregulation was present in 33 of 51 patients [10]. Pham and colleagues observed that COx was significantly higher when comparing non-survivors to survivors in patients after cardiac arrest [34]. Interestingly, there was no relationship between the absolute value of rSO_2_ and outcome in this cohort. Our research group recently conducted a prospective observational study of real-time cerebral autoregulation monitoring using COx in 20 patients with HIBI following cardiac arrest [28]. Importantly, we were also able to demonstrate that this technique is feasible as we generated U-shaped autoregulation curves and identified the optimal MAP in 19 of 20 patients. The mean optimal MAP (MAP_OPT_) was 75 mmHg (standard deviation (SD) 10). The mean percentage of time spent outside 5 mmHg from MAP_OPT_ was 47% (SD 21%). Dysfunctional autoregulation (percentage of time with COx at 0.3 or above) occurred in 11% (SD 17%) of measurement time [28]. Furthermore, we demonstrated an association with increased COx and hyperthermia, highlighting the importance of temperature regulation in cerebrovascular reactivity after HIBI [28]. These findings are consistent with dysfunctional cerebral autoregulation observed during hyperthermia (>38 °C) in patients with traumatic brain injury [35]. Although hyperthermia appears to be associated with dysfunctional cerebral autoregulation after brain injury, currently no definitive data suggest a difference in the preservation of autoregulation in patients managed with normothermia (36 °C) versus hypothermia (33 °C), making it a logical question for future research.

Significant work remains to delineate the role of COx-based autoregulation monitoring in adult HIBI patients. In traumatic brain injury, a similar Pearson correlation coefficient between MAP and intracranial pressure (PRx) can individualize cerebral perfusion pressure thresholds [36]. Investigators have demonstrated that perfusion within the intact zone of autoregulation as determined by PRx is associated with improved cerebral oxygenation [37]. Furthermore, a large observational outcome study has suggested that perfusion within 5 mmHg of the optimal cerebral perfusion pressure after traumatic brain injury is associated with improved long-term neurological outcomes [38]. With this in mind, further examining the relationship of individualized cerebral autoregulation thresholds and neurologic outcomes in patients with HIBI is a logical next step. Additionally, the changes that occur with autoregulation in adult HIBI patients and key physiologic modulators, including arterial carbon dioxide concentration, core body temperature, intravenous sedatives and vasoactive agents, are essential research questions. Finally, a comparison of the identification of optimal MAP using COx versus direct measures of CBF in an adult model of HIBI is imperative moving forward.

Although using cerebral autoregulation monitoring to titrate MAP is an intriguing therapeutic strategy, several limitations need to be addressed. Firstly, cardiac arrest often results in impaired left ventricular function, which may variably tolerate the increase in afterload from exogenous vasopressor administration for targeting right-shifted optimal MAP using COx. Furthermore, in patients who suffer a primary cardiac arrest, administration of vasopressors to increased MAP will increase ventricular wall stress and myocardial oxygen consumption and potentially extend the infarction core. High doses of vasopressors themselves have significant risks, including arrhythmias, increased pulmonary vascular permeability and myocardial, mesenteric and digital ischemia. Therefore, the risks associated with increased MAP targets must be weighed against the potential benefits of improved cerebral perfusion and oxygenation.

NIRS monitoring itself has significant limitations, notably the contamination of the rSO_2_ signal from both ambient light and extracranial blood flow, albeit, the precise proportion of signal interference from scalp blood flow is not well established [39]. Furthermore, skin moisture stemming from forehead perspiration can lead to inadequate adherence of NIRS probes and inaccurate signal [39]. It should be recognized that NIRS only samples the 1 to 2 cm of superficial frontal lobe tissue beneath the forehead and, as such, may not reflect regional differences in autoregulation in crucial cerebral structures particularly susceptible to injury after HIBI, including the grey–white matter interface, basal ganglia and hippocampi [39]. Finally, important physiologic variables such as acute fluctuations in hemoglobin concentration, arterial carbon dioxide and oxygen tension can influence the NIRS signal, thereby changing the rSO_2_ independently of fluctuations in MAP. This is a major pitfall in NIRS-based autoregulation monitoring in that the relationship between MAP and rSO_2_, which generates COx, can be altered by acute changes in these other physiologic variables.

## Conclusions

Autoregulation monitoring represents a recent advance and unique opportunity to further develop our understanding of the perturbations in the cerebrovascular hemodynamics in patients with HIBI. Cerebral autoregulation monitoring provides an opportunity to *individualize* perfusion targets rather than the traditional “one size fits all” approach.

## Abbreviations

CA: Cardiac arrest
CBF: Cerebral blood flow
CDO_2_: Cerebral oxygen delivery
HIBI: Hypoxic ischemic brain injury
ROSC: Return of spontaneous circulation
rSO_2_: Regional saturation of oxygen
